# Unsupervised Feature-Construction-Based Motor Fault Diagnosis

**DOI:** 10.3390/s24102978

**Published:** 2024-05-08

**Authors:** Tsatsral Amarbayasgalan, Keun Ho Ryu

**Affiliations:** 1 Electronics and Telecommunications Research Institute, Daejeon 34129, Republic of Korea; tsatsral@etri.re.kr; 2 Data Science Laboratory, Faculty of Information Technology, Ton Duc Thang University, Ho Chi Minh City 700000, Vietnam

**Keywords:** motor bearing fault, fault detection, neural network, vibration signal

## Abstract

Any bearing faults are a leading cause of motor damage and bring economic losses. Fast and accurate identification of bearing faults is valuable for preventing damaging the whole equipment and continuously running industrial processes without interruption. Vibration signals from a running motor can be utilized to diagnose a bearing health condition. This study proposes a detection method for bearing faults based on two types of neural networks from motor vibration data. The proposed method uses an autoencoder neural network for constructing a new motor vibration feature and a feed-forward neural network for the final detection. The constructed signal feature enhances the prediction performance by focusing more on a fault type that is difficult to detect. We conducted experiments on the CWRU bearing datasets. The experimental study shows that the proposed method improves the performance of the feed-forward neural network and outperforms the other machine learning algorithms.

## 1. Introduction

Motor failures are often associated with bearing damage. This can result in high levels of vibration that can disrupt production processes, cause motor malfunctions, and lead to economic losses. Therefore, it is important to detect bearing faults early by monitoring motor vibration signals to prevent more damage to the equipment and ensure uninterrupted industrial processes [1]. In general, running motor vibration signals are collected very quickly, and data-driven learning techniques have been increasingly used to diagnose motor health conditions from these signals.

The methods for detecting bearing faults can be classified as statistical-feature-based detectors and raw-signal-based detectors. Statistical-feature-based detectors first extract statistical features from time-domain signals or frequency-domain signals, such as the maximum value of amplitude, mean value of amplitude, and Kurtosis factor. These features are then used to detect the fault type [1,2,3,4,5,6,7]. However, the efficiency of statistical features may vary based on datasets and detection models, and it requires manual feature extraction for each specific case. In contrast, deep-learning-based methods do not require feature extraction because of their enormous learnable parameters. These methods learn useful features automatically without an additional feature pre-processing stage [8,9,10,11]. Deep-learning-based detectors significantly increase detection performance from raw signals, making them more suitable for bearing fault detection.

This study proposes an unsupervised neural-network-based signal extraction for improving bearing fault detection using autoencoder (AE) neural network (AENN) and feed-forward neural network (FFNN) models. However, the process of signal extraction is distinct from statistical-based methods and involves the use of self-supervised learning. The proposed method differs from existing motor fault detection methods. It improves the detection performance by focusing more on the fault type that is misclassified the most during the training process. Specifically, the performance of the FFNN model is enhanced through the extracted signal utilizing an unsupervised AE model. The AE model learns from vibration signals belonging to a fault type that was detected with more error than the other fault types. The most misclassified fault type for the AE model is chosen using the FFNN model on the original raw signals. In general, the structure of an AE model has the same number of neurons in the input and output layers, and its output is a reconstructed version of the input. The AE model learns by minimizing the variance between input and output values. In this study, we use the difference between input and output values on the AE model, known as reconstruction error (RE), as a new feature to reduce the detection error of the FFNN model. When the inputs are signals other than those used to construct the AE model, they are reconstructed with a high difference. This is because the AE model shows a high gap between the input and output when the input is unlearned types of signals. The main contributions of this study are as follows:

- The proposed method improves the detection performance of the FFNN model by signal extraction, which better distinguishes a fault type that is hard to detect;
- We evaluated the proposed method on open datasets collected with different horsepower. The proposed method gave a higher performance than the compared methods.
- The rest of this paper is organized as follows. Section 2 provides an overview of existing studies for motor bearing fault detection. The proposed method is detailed in Section 3. Section 4 demonstrates the experimental study, including experimental design, dataset, evaluation metrics, and detection performances of the compared methods. In Section 5, we conclude this study.

## 2. Related Work

It is beneficial to detect motor failures automatically and accurately to prevent future failures and financial losses due to sudden breakdowns and interruptions. As mentioned above, the utilization of machine learning algorithms to detect motor faults using signal data from motor vibration sensors is increasing. These methods typically build the motor fault detection model based on feature extraction and feature selection steps. Chuan et al. proposed a deep random forest fusion (DRFF) technique to diagnose gearbox faults using acoustic and vibratory signals [1]. First, they transformed signals from the gearbox by a wavelet packet transformer (WPT) and extracted features using two deep Boltzmann machines (DBMs). Finally, the random forest (RF) classifier fused the outputs of the two DBMs. The authors of [2] offered support vector machines (SVMs) and K-nearest neighbors (KNNs), and the bagged tree-based classifiers provided nearly 100 percent accuracy for motor fault diagnosis from both stator currents and vibration signals of motors. They extracted eight statistical features for the detection model: mean, median, standard deviation, and other criteria. These features were calculated from the results of two signal processing techniques: the matching pursuit (MP) and discrete wavelet transform (DWT). In [4], there were eight wavelet features extracted using a three-layer WPT transformer on the raw signal dataset to be used as the input to the classification model. Then, ensemble learning algorithms, such as adaptive boosting (AdaBoost) and RF, were suggested to diagnose crack faults in the presence of noise and small data. Another IoT-based ensemble algorithm was proposed in [5] to monitor the status of the induction motor from the motor vibration signal. Sundaram Buchaiah et al. [11] selected important features from 72 statistical features using the RF algorithm for bearing data. Then, they reduced the dimension of input features into two by dimension reduction techniques. Finally, the Bhattacharyya distance and SVM algorithms were used to verify fault diagnosis accuracy.

Recently, deep-learning-based methods have been used broadly for fault detection. The advantage of deep learning is that it does not require manual feature extraction. In [12], a deep neural network was proposed to detect multi-faults from raw sensor data without feature selection and signal processing. The authors of [9] used signals from multiple current sensors instead of vibration sensors. The current signal is accessible by low-cost sensors and not easily affected by interfering noise from the surrounding components. They diagnosed seven types of gearbox conditions, which were diagnosed by a two-dimensional convolutional neural network (CNN). Jong-Hyun Lee et al. [10] proposed the CNN model to develop a motor fault diagnosing system. It detects whether a motor condition is normal or faulty on the rotor and bearing from the vibration signals without signal preprocessing. Many studies have used the AE model for bearing fault detection [8,13,14,15,16]. The authors of [8] proposed a stacked denoising autoencoder (SDAE)-based fault detector from motor vibration signals. First, the Fourier transform produced frequency-domain signals for use as input to the SDAE. Then, several unsupervised AE models were used to extract features. The input layer of the next AE used the encoder layer of the above-level AE, and the final AE’s encoder layer was used for classification with the Softmax function. In the stacked denoising autoencoder (SDA) was investigated for the fault diagnosis of rotary machinery components when signals have ambient noise and working condition fluctuations. They stacked three AE models to obtain high-level feature representations to improve classification robustness. The authors of proposed an ensemble of deep autoencoders (EDAE) for feature learning from the vibration signals and fault diagnosis. EDAEs were constructed using fifteen AE models with various activation functions. The features from the last AE model were fed into the Softmax classifier for fault recognition.

Many machine-learning-based studies extracted fault detection features manually, based on their domain knowledge, or using some statistical approaches, such as kurtosis and skewness. In contrast, deep-learning-based methods do not require feature extraction because of their enormous learnable parameters. The proposed method in this study differs from existing studies by extracting an additional feature using the advantage of self-supervised deep neural networks. It focuses on the most misclassified fault type using the extracted signal feature to improve the fault detection model. We extract a new signal feature by reconstruction error of the input signal on the AE model that learns from only the fault type, which is the most difficult type to detect. Therefore, the value of the reconstruction error of the input signal belonging to the fault type used in the training of the AE model will be lower than the reconstruction errors of other types of signals. This characteristic of the AE trained from the single type of fault enhances distinguishing faults from the vibration signal data.

## 3. Proposed Method for Bearing Fault Detection

**Figure 1:**
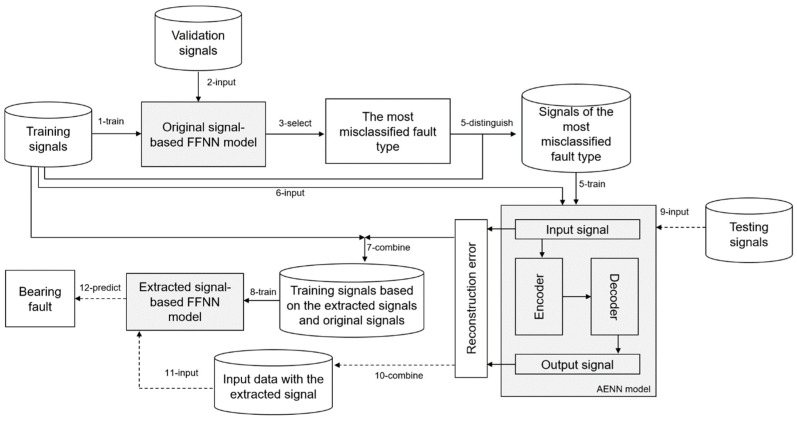
General architecture of the proposed detection method for bearing faults; FFNN: feed-forward neural network, AENN: autoencoder neural network.

This section describes the training and testing processes of the proposed method to predict bearing faults as shown in Figure 1. The solid lines indicate the training steps that build the detection model for the bearing faults, and the dashed lines represent the testing process of fault detection on unseen signals.

The proposed method addresses the most challenging fault type for reducing detection error. Therefore, we first train an initial FFNN model from the original training signals (step 1). This initial FFNN model is used to determine the most challenging fault type from the evaluation of the validation signals (steps 2 and 3). Then, signals belonging to the selected (determined as challenging) fault type are distinguished from the whole training signals in step 4. Next, the AENN model learns from the selected type of faulty signals for feature extraction in step 5. In step 6, to generate a new feature based on the initial training signals marked as “A” in Figure 1, we feed the signals to the AENN model and obtain reconstruction errors from the AENN model. To create the final training dataset, we combine the initial training signals (marked as “A”) with the received reconstruction errors in step 7. The initial training dataset consists of input signals with 120 vibration points. After step 7, the length of the input signal becomes 121. In the last step of the training process, the second FFNN model is trained from the final prepared training signals (marked as “B”) for further detection.

The testing process (detect faults from the unseen signals) is demonstrated in steps 9–12. Before detecting bearing fault by the final FFNN model, the reconstruction error of the input signal is obtained from the AENN model (step 9). In steps 10–12, the final FFNN model detects the bearing health condition from the combination of unseen input signal and its reconstruction error.

In this study, we utilize an AE neural network model to generate a new feature from the input signal, which will enhance the detection model based on the FFNN with three hidden layers. Our proposed method involves simpler model architectures than CNNs, which are usually more effective in dealing with high-dimensional data, such as images. Therefore, we have employed the reconstruction error of the input signals on the AENN model with a single hidden layer to improve the feature representation of the FFNN.

**Figure 2:**
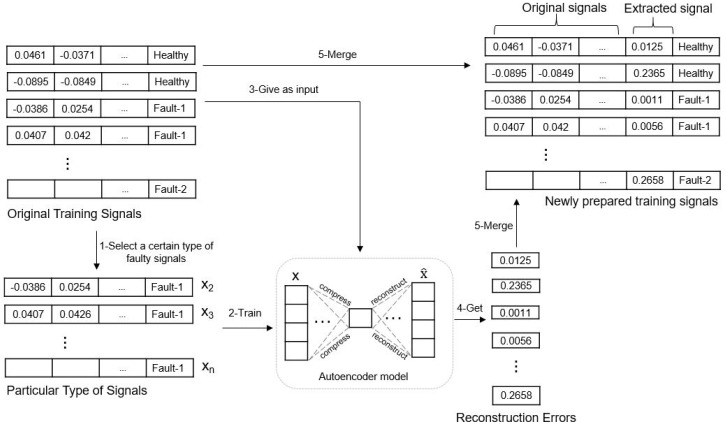
Example of the AE model preparation in the proposed method.

Figure 2 shows an example of how the AE model is constructed in the proposed method. First, we separate the particular type of signals from the whole signals based on the detection results from the baseline FFNN model (original signal-based FFNN model) shown in Figure 1. For instance, the AE model trained on the selected Fault-1-type signals in Figure 2. The bottleneck hidden layer of the AE model in the proposed method has 60 neurons and transformed the received values from the preceding layer by the rectified linear unit (ReLU) activation function. To better distinguish Fault-1 from other bearing faults, a new signal feature is extracted by giving a training signal to the prepared AE model and calculating the RE. As a result, the final training signals are made by combining the original signals with the reconstruction error of the input signal.

The AE is a symmetric neural network where the numbers of neurons in the input and output layers are the same. It learns the data pattern by reducing the input dimension and then reconstructing the input from the reduced dimensional space. Due to its structure, it has been used in dimension reduction, data denoising, and synthetic data generation. For example, a bottleneck layer of the AE model that is a compressed representation of its input is used in dimension reduction. In the training process of the AE, if inputs are data with noise and outputs are the original data, the AE can be applied for data denoising. In this paper, we use RE, which is a variance between the input and output layers of the AENN model, to emphasize a particular type of motor fault. RE is defined as follows (1):

(1) $$RE=\frac{\sum_{j=1}^{k}{\left(x_{j}-{\hat{x}}_{j}\right)}^{2}}{k}$$

where k is the number of neurons in the input and output layers of the AE, $x_{j}$ is the j-th neuron in the input layer, and ${\hat{x}}_{j}$ is the corresponding neuron with the reconstructed value of $x_{j}$.

The proposed method predicts motor bearing faults by the FFNN model. The neural network was first introduced in 1943 [17] and has been successfully applied in various domains, such as image processing [18], natural language processing [19], and predicting motor faults [20]. The FFNN is constructed by fully connected (dense) layers that are simpler in architecture to compare convolutional neural networks (CNNs). The fully connected layers consist of neurons, and every neuron connects to all neurons in the descendant layer. CNNs are typically more effective when dealing with high-dimensional data like images in which local patterns and spatial relationships are important. Fully connected layers process the entire input signal as a single vector, allowing them to capture the relevant information that spreads across the entire signal rather than being localized in specific regions. In particular, an underlying pattern of the CWRU dataset was effectively captured with an accuracy exceeding 90% by a simple neural network with fully connected dense layers. For this study, we propose a simple fully connected feed-forward neural network with three hidden layers.

**Figure 3:**
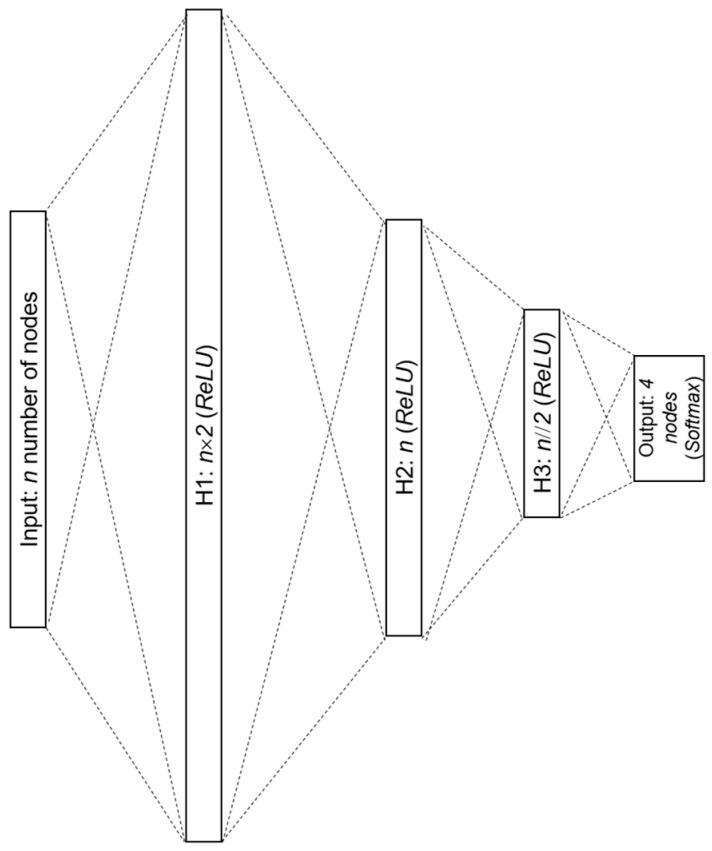
Structure of the FFNN model used in the proposed method; *n* is the number of input neurons, H1–H3 are the hidden layers; y is the number of output neurons.

The proposed FFNN has three hidden layers, as shown in Figure 3. Neurons in each hidden layer transform their received value from the preceding layer by the ReLU activation function. The output layer uses the Softmax activation function to predict motor conditions; it returns the probability of each motor condition as a value from 0 to 1, and the sum of all probabilities is equivalent to 1. From the result outputs, the high-probability bearing fault is chosen as the final detection result.

The FFNN model is trained on the prepared training signals by combining the original and extracted signals. It can improve the detection performance by using the extracted signal based on the most misidentified fault type.

## 4. Experimental Study

### 4.1. Dataset

The Case Western Reserve University (CWRU) bearing dataset [21] is a popular benchmark dataset in machinery fault diagnosis. This dataset is relatively large, containing vibration signals gathered from bearings under various fault conditions, including inner race, outer race, and ball faults, as well as healthy conditions. There are several reasons to apply data-driven learning methods, especially neural networks, to this dataset for monitoring motor health conditions. Neural networks handle large datasets and process the entire dataset without manual operation. Moreover, neural networks are robust to noise from variations in operating conditions and other sources of variability commonly encountered in real-world industrial environments. The fault conditions represented in the CWRU dataset are common in industrial machinery, such as rotating equipment with rolling element bearings. By developing machine learning models on this dataset, researchers aim to improve the reliability and efficiency of machinery condition monitoring systems in real-world applications. We used the CWRU bearing dataset to evaluate fault detection methods.

**Figure 4:**
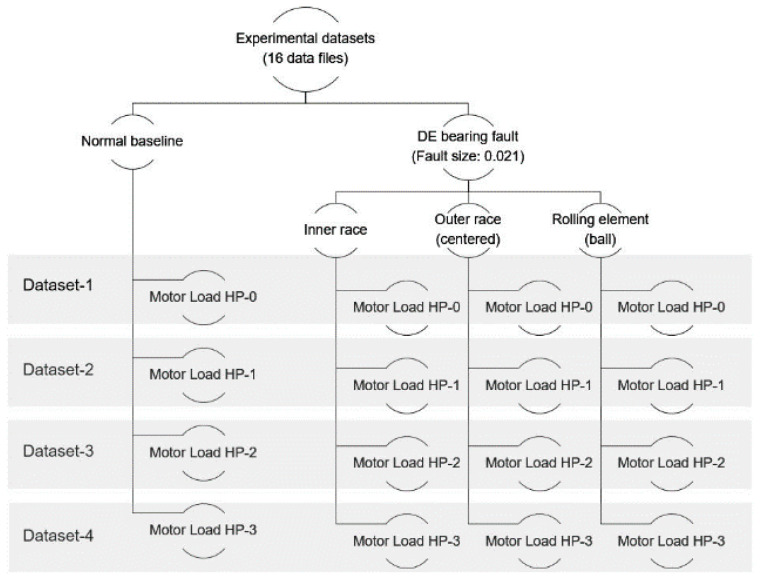
Experimental datasets; DE: drive end.

**Figure 5:**
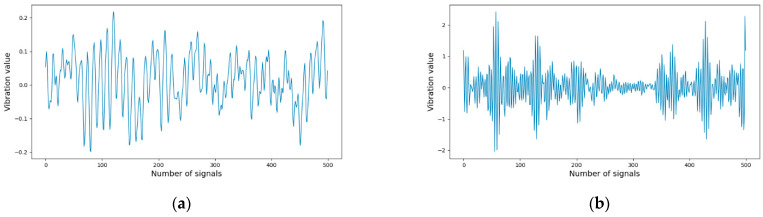

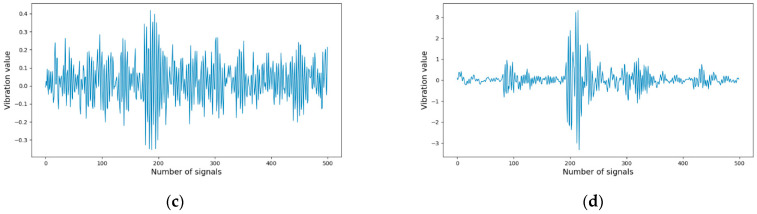
Example of the first 500 vibration signals for each bearing condition. (**a**) File: 98 (normal); (**b**) File: 210 (inner race fault); (**c**) File: 223: (ball fault); (**d**) File: 235 (outer race centered fault).

The vibration signals were recorded from healthy bearings and three types of faulty bearings, including the inner raceway, rolling element (ball), and outer raceway, each with a failure diameter of 0.021 inches, with motor speeds from 0 to 3 horsepower. We utilized drive-end (DE) bearing vibration signals in the experimental study. There were four datasets prepared for the experimental study. Each dataset consists of four files for normal bearing conditions and three kinds of faulty bearing conditions, as shown in Figure 4. Table 1 shows the data files used in the experimental study. Figure 5 demonstrates an example of the vibration signals of bearing health conditions.

### 4.2. Compared Detection Methods

The proposed detection method for bearing faults has been compared with machine learning algorithms, including KNN, SVM, AdaBoost, decision tree (DT), naïve Bayes (NB), and RF. We implemented the compared detection models in Python using the Scikit-learn package [22] for machine learning classifiers and the Keras library [23] for deep neural networks. The parameter configurations of the compared algorithms are shown in Table 2. We trained several models from each algorithm using varying configurations. For instance, we trained KNN-based models with different numbers of neighbors ranging between 3 and 25. To compare the KNN-based detection model with others, we selected the best-performing model on test datasets from these KNN models. Table 3 shows the configurations of the models in the proposed method.

**Figure 6:**
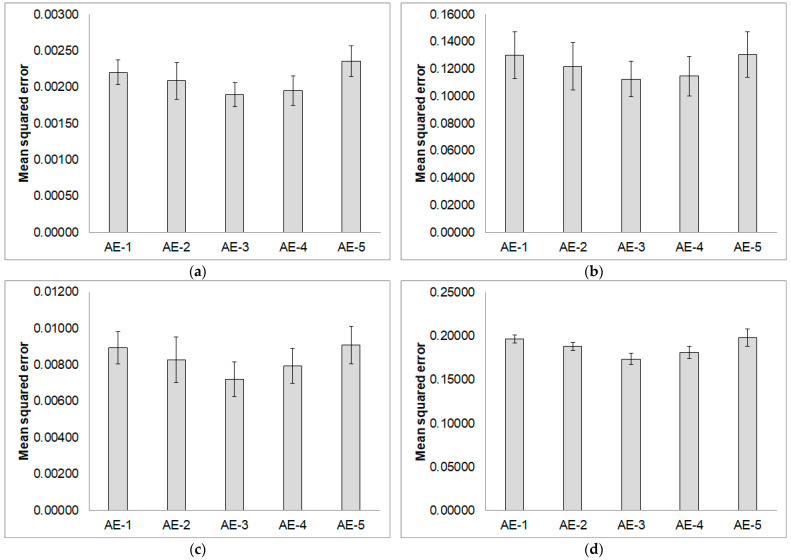
Average training errors with standard deviations of AE models on datasets 1–4. (**a**) AE models learned from the normal signals; (**b**) AE models learned from the inner race fault signals; (**c**) AE models learned from the ball fault signals; (**d**) AE models learned from the outer race centered fault signals. AE-1: layers [120, 60, 30, 15, 30, 60, 120]; AE-2: layers [120, 60, 30, 60, 120]; AE-3: layers [120, 60, 120]; AE-4: layers [120, 30, 120]; AE-5: layers [120, 15, 120].

The proposed method employs AE and FFNN models to detect bearing faults. We generate an additional feature from the input signal via the AE model to enhance the detection performance of the FFNN model. This additional feature is generated by the reconstruction error of the input signal on the AE model. We train AE models with different structures on signals of all types of bearing conditions to select the AE model with the lowest training error. Figure 6 represents the average mean squared error of the trained AE models on datasets 1–4 shown in Table 1. The AE-3 model with a single hidden layer of 60 nodes gave the smallest average error for all bearing health conditions. Therefore, the proposed method used AE-3 for extracting the RE-based feature. The structure of the AE-3 model has an input layer with 120 nodes, a hidden layer (latent space) with 60 nodes, and an output layer with 120 nodes.

**Figure 7:**
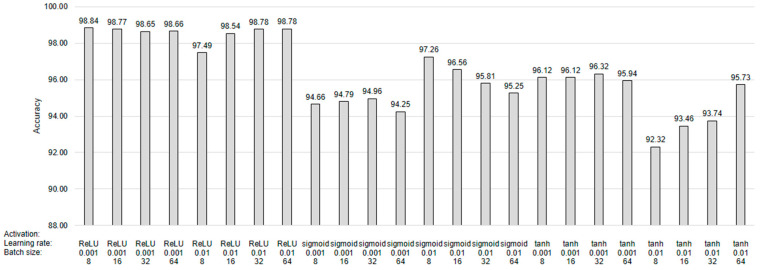
Average validation accuracy of FFNN models with different hyperparameter configurations on experimented datasets.

For the FFNN model used in the proposed method, we trained various FFNN models to choose the appropriate hyperparameter configurations. Figure 7 shows the average validation accuracy of the baseline FFNN and the proposed FFNN models on datasets 1–4. We used 90% of the dataset for training, and the remaining 10% was used for validation. The learning rate of 0.001 and batch size of 8 with the ReLU activation function showed higher accuracy than the other configurations.

### 4.3. Experimental Results

First, the baseline FFNN is learned from the original training signals. We found the most misclassified type of bearing fault from the training dataset using the baseline FFNN model to train the AE model on the most challenging fault type. Then, the proposed FFNN model was trained on the original and AE-based extracted signals. Table 4 shows the confusion matrix of baseline FFNN models on the validation set, which is 10% of the training signals. In datasets 1–3, the outer race fault was the most misclassified. However, the ball fault in Dataset-4 was more incorrectly detected than other bearing faults.

We compared the proposed detection model and baseline FFNN model to demonstrate how the proposed method improved the detection performance of the baseline FFNN model. We trained four baseline models using the original input signals of datasets 1–4 separately. Then, the proposed FFNN models were trained by the original and extracted input signals. For signal extraction for the proposed FFNN, we selected AE models learned from the most misclassified motor condition signals on each dataset based on the confusion matrix shown in Table 4. Each model was tested on three datasets, and the final performance was averaged in Table 5. We can see that the proposed AE-based FFNN models outperform the baseline FFNN models based on the initial signals without the extracted feature, and their average evaluation metrics were higher than the baseline FFNN models. Moreover, the recall and f-measure measurements of baseline FFNN models were improved by using the extracted feature from the AE model, and its average values increased by more than 1% by the proposed method.

**Figure 8:**
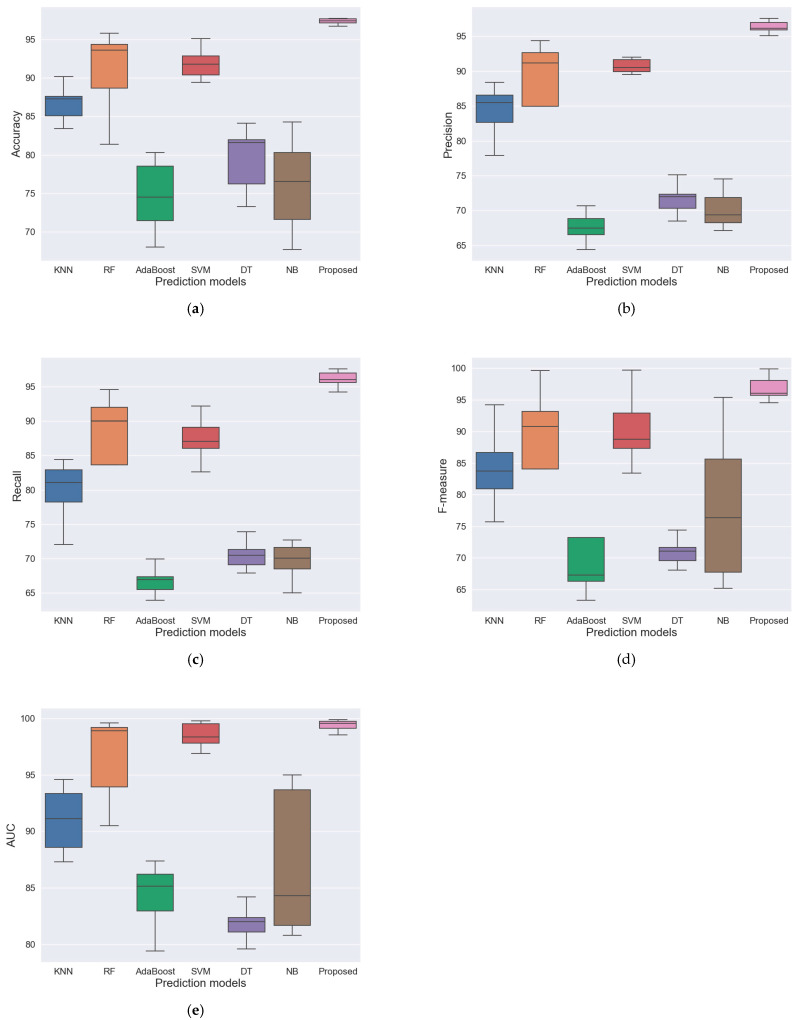
Performance distribution of the compared models across the experimented datasets. (**a**) Box plot of the accuracy; (**b**) box plot of the precision; (**c**) box plot of the recall; (**d**) box plot of the f-measure; (**e**) box plot of the AUC.

Finally, we compared six machine learning classification models with the proposed method, including KNN, RF, AdaBoost, SVM, NB, and DT. We experimented with four different datasets gathered by different shaft speeds. Four models were trained for each algorithm using datasets listed in Table 1 for performance evaluation. Then, each model was tested on three untrained datasets. For instance, a model trained on Dataset-1 with a shaft speed of 1797 rpm was tested on Dataset-2, Dataset-3, and Dataset-4 with shaft speeds of 1772, 1750, and 1730 rpm, respectively. Table 6 shows the average performances, and detailed performances are represented in Appendix A. The proposed method showed more stable results than the compared methods on the datasets collected at different shaft speeds, as shown in Figure 8.

Table A1, Table A2, Table A3 and Table A4 show the comparison of the proposed AE-FFNN model and other machine-learning-based predictive models. We configured the input parameters of the particular machine learning models differently based on the dataset. The best values for the input parameters were selected based on the training performance. As a result of these comparisons, the proposed detection method performed better than the compared individual predictive methods.

Table A1 shows the testing results on datasets 2–4. All models learned from Dataset-1. The RF model showed higher results than the machine-learning-based models on each testing dataset. However, the proposed method outperformed the RF by increasing the average accuracy, precision, recall, f-measure, and AUC values on datasets 2–4 by 3.184%, 4.154%, 5.590%, 4.901%, and 0.509%, respectively.

For machine-learning-based compared detection models learned from Dataset-2, the RF model performed better than others on all testing datasets, including Dataset-1, Dataset-3, and Dataset-4. However, the proposed method outperformed its accuracy by 1.762%, precision by 3.023%, recall by 3.382%, f-measure by 4.024%, and AUC by 2.815% on average, as shown in Table A2.

Table A3 shows the results of the compared models trained on Dataset-3, and evaluated on datasets 1, 2, and 4. As a result of the compared models, except for the proposed model, RF and SVM models showed comparable higher results than others. However, the proposed method was superior to these models.

In Table A4, we showed the results on datasets 1–3 for detection models learned from Dataset-4. We can see that the proposed method achieves the best performance for predicting bearing faults. Its average accuracy, precision, recall, f-measure, and AUC on test datasets reached 98.18%, 96.651%, 96.007%, 95.547%, and 96.699%, respectively. These are higher than the RF model by 4.184%, 5.64%, 5.947%, and 3.766%.

Figure 8 represents distributions of detection performances across all experimented datasets of the compared models learned from datasets 1–4 by the box plot diagram. According to all evaluation measurements, RF and SVM models showed comparable results with the proposed method by giving performances that were higher than 80%. We can see that the performance of the SVM model was less spread out than the RF model, and its value was relatively lower. However, the proposed method performed better than other models according to all measurements by showing the shortest performance distribution with the highest value.

## 5. Conclusions

In this study, we proposed a method for bearing fault detection from motor vibration signals based on two different types of neural networks, such as the AENN for feature extraction and the FFNN for detection. The AENN is a type of neural network that reconstructs a given input into its output as similarly as possible. Mainly, it is used for data denoising by reconstructing noisy data or for generating synthetic data from its learned distribution. In the proposed method, we used AENN’s input–output difference to extract the input feature for the bearing fault detection model. By training the AENN model on only the most complicated signal types instead of all signal types, we were able to use its RE to make a feature to help distinguish fault types. In other words, the AENN model reconstructs a given input signal to the output with less loss when the input is of a learned signal type rather than its unlearned signal type.

The AENN model is learned from signals of a single type of fault to emphasize that type of fault over others. However, the detection models were designed with four outputs for ball fault, inner race fault, outer race fault, and normal condition of motor bearings. The limitation of the proposed model is that it can improve the fault detection FFNN model based on the additional generated feature that can emphasize only one fault type.

We evaluated the proposed method on the CRWU bearing open dataset and compared it with six machine-learning-based models. The presented method successfully enhanced the detection performances of the FFNN by focusing on the most challenging bearing fault type to detect using the AENN model. It increased average values of the recall and f-measure of the baseline FFNN by more than 1% on the experimented datasets. Moreover, the proposed method outperformed the average accuracy, precision, recall, f-measure, and AUC of the KNN, RF, AdaBoost, SVM, DT, and NB models in Table A1, Table A2, Table A3 and Table A4 by (10.430, 10.474, 14.298, 15.454, 10.919), (2.688, 3.632, 4.606, 4.771, 2.544), (18.013, 25.124, 29.490, 29.479, 21.619), (4.105, 5.474, 7.356, 7.971, 4.236), (16.480, 21.790, 25.573, 25.679, 21.561), and (15.171, 24.724, 28.381, 28.413, 18.236), respectively.

## Figures and Tables

**Table 1:** Data files of the experimental datasets.

Prepared Dataset	Motor Load hp	Shaft Speed rpm	File Number	Fault Types	Fault Size	# Samples	Min	Max	Avg	Stdev
Dataset-1	HP-0	1797	97	Normal	*	243,938	−0.287	0.311	0.013	0.073
			222	Ball	0.021	121,991	−1.618	1.660	0.021	0.134
			209	Inner race	0.021	122,136	−3.372	3.788	0.014	0.525
			234	Outer race centered	0.021	122,426	−6.412	6.653	0.005	0.583
Dataset-2	HP-1	1772	98	Normal	*	483,983	−0.346	0.318	0.013	0.065
			223	Ball	0.021	121,701	−1.414	1.475	0.004	0.129
			210	Inner race	0.021	121,556	−3.284	3.686	0.003	0.442
			235	Outer race centered	0.021	122,281	−6.653	6.653	0.003	0.57
Dataset-3	HP-2	1750	99	Normal	*	485,063	−0.327	0.359	0.012	0.063
			224	Ball	0.021	122,136	−0.531	0.646	0.005	0.107
			211	Inner race	0.021	121,846	−3.124	3.623	0.003	0.489
			236	Outer race centered	0.021	121,991	−6.653	6.653	0.004	0.559
Dataset-4	HP-3	1730	100	Normal	*	485,643	−0.306	0.284	0.012	0.065
			225	Ball	0.021	122,136	−0.493	0.576	0.004	0.118
			212	Inner race	0.021	121,991	−3.087	3.615	0.003	0.449
			237	Outer race centered	0.021	121,991	−6.113	6.207	0.003	0.561

Avg: average value; Max: maximum value; Min: minimum value; Stdev: standard deviation. Please note that the symbol “*” indicates that the fault size is irrelevant.

**Table 2:** Input parameters of the experimented classifiers.

Classifiers	Configured Values
K-Nearest Neighbors	The number of neighbors was configured from 3 to 25.
Support Vector Machine	The linear, polynomial, radial basis function (rbf), and sigmoid kernels were used.
Adaptive Boosting	The number of estimators was configured from 10 to 150.
Decision Tree	Classification criteria were configured by “gini” and “entropy”.
Random Forest	The number of trees was configured from 10 to 150.

**Table 3:** Configurations of the models in the proposed method: the proposed method consists of two neural network models for reconstruction-error-based feature generation and fault detection.

Configurations	FFNN	AENN
Layer: (neurons)	Input: (121) Hidden: (242, 121, 60) Output: (4)	Input: (120) Hidden: (60) Output: (120)
Layer: (activation function)	Hidden: (ReLU) Output: (Softmax)	Hidden: (ReLU)
Learning rate	0.001	0.001
Optimizer	Adam [24]	Adam [24]
Loss function	Categorical Cross-Entropy	Mean Squared Error
Batch size	8	8
Epochs	500 early stopping [25] of 100	500

**Table 4:** Confusion matrix of the baseline FFNN models on datasets 1–4.

		Actual						Actual			
**Predicted**	**Dataset-1**	Normal	Ball fault	Inner race fault	Outer race fault	**Predicted**	Dataset-2	Normal	Ball fault	Inner race fault	Outer race fault
	Normal	201	0	0	1		Normal	395	0	0	0
	Ball fault	0	111	1	1		Ball fault	0	116	1	1
	Inner race fault	0	1	91	2		Inner race fault	0	1	96	3
	Outer race fault	0	1	0	99		Outer race fault	0	1	1	93
	Missed	0	2	1	4		Missed	0	2	2	4
		**Actual**						**Actual**			
**Predicted**	Dataset-3	Normal	Ball fault	Inner race fault	Outer race fault	**Predicted**	Dataset-4	Normal	Ball fault	Inner race fault	Outer race fault
	Normal	397	0	0	0		Normal	403	0	0	0
	Ball fault	0	95	0	1		Ball fault	0	96	0	3
	Inner race fault	0	0	105	6		Inner race fault	0	0	104	0
	Outer race fault	0	1	0	104		Outer race fault	0	6	0	98
	Missed	0	1	0	7		Missed	0	6	0	3

**Table 5:** Comparison between the baseline FFNN models and the proposed AE-FFNN models on datasets 1–4.

Performance	Models Were Trained on the Dataset-1, and Tested on the Datasets 2–4		Models Were Trained on the Dataset-2, and Tested on the Dataset 1, 3, 4		Models Were Trained on the Dataset-3, and Tested on the Datasets 1, 2, 4		Models Were Trained on the Dataset-4, and Tested on the Datasets 1–3	
	Baseline	Proposed	Baseline	Proposed	Baseline	Proposed	Baseline	Proposed
Accuracy	96.694 (0.003)	97.484 (0.002)	97.724 (0.007)	98.284 (0.004)	96.502 (0.012)	97.489 (0.006)	95.526 (0.018)	96.489 (0.012)
Precision	95.045 (0.004)	95.887 (0.002)	96.833 (0.004)	97.462 (0.002)	95.064 (0.009)	96.296 (0.005)	94.974 (0.012)	95.573 (0.009)
Recall	94.237 (0.006)	95.69 (0.004)	96.574 (0.005)	97.404 (0.002)	94.722 (0.011)	96.173 (0.005)	93.339 (0.017)	94.726 (0.01)
F-measure	94.49 (0.006)	95.735 (0.004)	96.691 (0.005)	97.42 (0.002)	94.849 (0.01)	96.203 (0.005)	93.947 (0.016)	95.048 (0.01)
AUC	99.717 (0.001)	99.776 (0)	99.746 (0.001)	99.835 (0.001)	99.465 (0.002)	99.513 (0.002)	99.578 (0.002)	99.606 (0.001)

**Table 6:** Average performance of the compared models.

Performance	KNN	RF	AdaBoost	SVM	DT	NB	Proposed
Accuracy	87.200	94.408	77.450	91.975	80.133	78.233	**97.559**
Precision	86.142	92.425	67.750	91.025	71.417	68.008	**96.444**
Recall	79.425	91.142	66.408	87.383	70.300	68.425	**96.189**
F-measure	81.575	91.725	66.892	88.792	70.742	66.967	**96.274**
AUC	91.867	99.283	86.283	99.125	81.833	93.433	**99.699**

## Data Availability

Data are available at https://engineering.case.edu/bearingdatacenter/download-data-file (2 October 2023).

## Appendix A

Table A1, Table A2, Table A3 and Table A4 show the comparison of the proposed AE-FFNN model and other machine-learning-based predictive models.

**Table A1:** Comparison of the experimented fault detection models learned from Dataset-1.

Test Dataset	Performance	KNN	RF	AdaBoost	SVM	DT	NB	Proposed/AENN Learned from the Outer Race Fault/
Dataset-2	Accuracy	87.300	93.600	77.500	91.300	81.800	81.400	97.739
	Precision	86.100	90.800	64.700	90.700	71.900	70.500	96.128
	Recall	77.800	88.900	62.200	84.800	70.500	68.100	96.168
	F-measure	80.200	89.800	63.300	87.200	71.100	67.700	96.133
	AUC	91.200	99.200	83.600	99.100	82.200	93.400	99.776
Dataset-3	Accuracy	85.100	94.000	79.800	90.400	81.700	79.900	97.518
	Precision	84.000	92.000	69.300	90.100	72.300	67.200	95.915
	Recall	73.900	89.600	65.700	83.200	70.400	65.000	95.724
	F-measure	77.300	90.700	67.300	86.100	71.100	65.200	95.794
	AUC	88.800	99.300	83.600	99.600	82.000	94.200	99.804
Dataset-4	Accuracy	83.900	95.300	80.300	92.300	80.200	84.300	97.196
	Precision	82.200	92.400	68.400	92.000	69.700	73.300	95.620
	Recall	72.000	91.800	66.600	86.600	68.900	72.700	95.178
	F-measure	75.700	92.000	67.400	88.900	68.900	71.400	95.276
	AUC	87.500	99.300	85.300	99.600	81.100	95.000	99.748

**Table A2:** Comparison of the experimented fault detection models learned from Dataset-2.

Test Dataset	Performance	KNN	RF	AdaBoost	SVM	DT	NB	Proposed/AENN Learned from the Outer Race Fault/
Dataset-1	Accuracy	87.500	94.300	72.000	90.400	76.900	69.100	97.699
	Precision	88.200	93.400	66.700	90.400	72.500	63.800	97.130
	Recall	84.400	92.900	67.100	88.000	71.600	64.800	97.127
	F-measure	85.100	93.100	66.600	88.700	71.900	63.400	97.125
	AUC	94.600	99.400	86.000	99.500	82.000	90.800	99.707
Dataset-3	Accuracy	89.600	95.800	80.300	92.800	84.100	80.600	98.590
	Precision	88.400	94.300	70.700	90.900	75.100	67.100	97.573
	Recall	81.800	92.700	67.100	87.500	73.900	66.600	97.539
	F-measure	83.600	93.400	68.600	89.000	74.400	66.200	97.527
	AUC	93.500	99.600	85.000	99.800	84.200	94.500	99.904
Dataset-4	Accuracy	87.300	96.800	80.800	95.200	80.800	84.800	98.563
	Precision	85.000	94.800	68.900	93.100	70.200	72.400	97.683
	Recall	77.900	94.400	69.200	91.500	69.000	73.800	97.546
	F-measure	80.300	94.600	68.600	92.200	69.500	71.900	97.607
	AUC	91.600	99.600	88.000	99.700	81.300	95.300	99.894

**Table A3:** Comparison of the experimented fault detection models learned from Dataset-3.

Test Dataset	Performance	KNN	RF	AdaBoost	SVM	DT	NB	Proposed/AENN Learned from the Outer Race Fault/
Dataset-1	Accuracy	86.500	93.300	71.700	89.400	74.300	65.400	96.716
	Precision	86.200	92.100	67.400	89.500	69.500	62.500	96.059
	Recall	83.100	91.600	67.300	86.700	69.100	63.400	95.910
	F-measure	83.900	91.800	67.300	87.400	69.300	61.200	95.961
	AUC	93.300	99.000	86.900	98.100	80.400	90.500	99.347
Dataset-2	Accuracy	90.200	94.500	77.000	94.000	82.500	79.900	97.527
	Precision	87.400	91.400	64.400	92.000	72.100	68.600	95.875
	Recall	82.900	90.400	63.900	89.600	71.200	70.600	95.707
	F-measure	84.200	90.900	64.000	90.700	71.600	67.800	95.733
	AUC	94.000	99.200	87.400	98.200	82.800	93.400	99.430
Dataset-4	Accuracy	90.100	96.500	79.400	96.900	81.800	83.800	98.224
	Precision	87.700	93.900	68.000	95.100	71.400	71.700	96.953
	Recall	82.800	93.900	67.600	94.500	70.800	74.500	96.901
	F-measure	83.900	93.900	67.500	94.700	71.100	71.500	96.914
	AUC	94.200	99.500	89.700	99.500	82.400	95.400	99.764

**Table A4:** Comparison of the experimented fault detection models learned from Dataset-4.

Test Dataset	Performance	KNN	RF	AdaBoost	SVM	DT	NB	Proposed/AENN Learned from the Ball Fault/
Dataset-1	Accuracy	83.400	91.100	71.500	86.100	73.300	67.600	95.398
	Precision	84.900	90.300	67.000	87.100	68.500	63.000	95.064
	Recall	79.300	88.900	66.700	82.600	67.900	64.200	94.247
	F-measure	81.200	89.500	66.800	83.400	68.100	62.400	94.539
	AUC	91.100	98.800	85.400	98.200	79.600	90.600	99.455
Dataset-2	Accuracy	87.600	93.600	78.100	92.300	81.500	80.700	97.358
	Precision	86.300	91.000	66.100	90.300	70.600	68.300	96.132
	Recall	78.400	88.800	64.800	86.500	68.900	69.200	95.393
	F-measure	81.600	89.900	65.400	88.200	69.700	67.700	95.677
	AUC	91.100	99.100	87.400	98.500	81.300	93.500	99.679
Dataset-3	Accuracy	87.900	94.100	81.000	92.600	82.700	81.300	98.181
	Precision	87.300	92.700	71.400	91.100	73.200	67.700	97.199
	Recall	78.800	89.800	68.700	87.100	71.400	68.200	96.826
	F-measure	81.900	91.100	69.900	89.000	72.200	67.200	97.002
	AUC	91.500	99.400	87.100	99.700	82.700	94.600	99.881
